# The relationship of oral health literacy with oral health-related quality of life in a multi-racial sample of low-income female caregivers

**DOI:** 10.1186/1477-7525-9-108

**Published:** 2011-12-01

**Authors:** Kimon Divaris, Jessica Y Lee, A Diane Baker, William F Vann

**Affiliations:** 1 Department of Pediatric Dentistry. 228 Brauer Hall, CB#7450, UNC School of Dentistry. University of North Carolina at Chapel Hill. Chapel Hill. North Carolina, 27599, USA; 2 Department of Epidemiology. 228 Brauer Hall, CB#7450, UNC School of Dentistry. University of North Carolina at Chapel Hill. Chapel Hill. North Carolina, 27599, USA; 3 Department of Health Policy and Management. CB#7411. University of North Carolina at Chapel Hill. Chapel Hill. North Carolina, 27599, USA

**Keywords:** oral health literacy, oral health-related quality of life, OHIP-14, racial differences, effect measure modification

## Abstract

**Background:**

To investigate the association between oral health literacy (OHL) and oral health-related quality of life (OHRQoL) and explore the racial differences therein among a low-income community-based group of female WIC participants.

**Methods:**

Participants (N = 1,405) enrolled in the Carolina Oral Health Literacy (COHL) study completed the short form of the Oral Health Impact Profile Index (OHIP-14, a measure of OHRQoL) and REALD-30 (a word recognition literacy test). Socio-demographic and self-reported dental attendance data were collected via structured interviews. Severity (cumulative OHIP-14 score) and extent of impact (number of items reported fairly/very often) scores were calculated as measures of OHRQoL. OHL was assessed by the cumulative REALD-30 score. The association of OHL with OHRQoL was examined using descriptive and visual methods, and was quantified using Spearman's *rho *and zero-inflated negative binomial modeling.

**Results:**

The study group included a substantial number of African Americans (AA = 41%) and American Indians (AI = 20%). The sample majority had a high school education or less and a mean age of 26.6 years. One-third of the participants reported at least one oral health impact. The OHIP-14 mean severity and extent scores were 10.6 [95% confidence limits (CL) = 10.0, 11.2] and 1.35 (95% CL = 1.21, 1.50), respectively. OHL scores were distributed normally with mean (standard deviation, SD) REALD-30 of 15.8 (5.3). OHL was weakly associated with OHRQoL: prevalence *rho *= -0.14 (95% CL = -0.20, -0.08); extent *rho *= -0.14 (95% CL = -0.19, -0.09); severity *rho *= -0.10 (95% CL = -0.16, -0.05). "Low" OHL (defined as < 13 REALD-30 score) was associated with worse OHRQoL, with increases in the prevalence of OHIP-14 impacts ranging from 11% for severity to 34% for extent. The inverse association of OHL with OHIP-14 impacts persisted in multivariate analysis: Problem Rate Ratio (PRR) = 0.91 (95% CL = 0.86, 0.98) for one SD change in OHL. Stratification by race revealed effect-measure modification: Whites--PRR = 1.01 (95% CL = 0.91, 1.11); AA--PRR = 0.86 (95% CL = 0.77, 0.96).

**Conclusions:**

Although the inverse association between OHL and OHRQoL across the entire sample was weak, subjects in the "low" OHL group reported significantly more OHRQoL impacts versus those with higher literacy. Our findings indicate that the association between OHL and OHRQoL may be modified by race.

## Background

The importance of subjective measures of oral health is well-recognized in dental research [1-3]. Theoretical models have provided the framework that links clinical conditions with patient perceptions and impacts on their oral health-related quality of life (OHRQoL) [4,5]. Evidence shows that individuals' perceptions of their dental condition is closely related to OHRQoL, [6] and may confer greater impacts than the actual clinical conditions [1]. The United States (US) Surgeon General's report on *Oral Health in America *underscores and emphasizes the importance of OHRQoL, and its improvement on a population-level is defined as a goal [7]. For these reasons, subjective oral health (SOH) instruments have been used to capture the multi-dimensional concept of OHRQoL [8,9] and are used to quantify patient outcome experiences, monitor oral health status on national level, and identify dental public health goals [10,11].

During this past decade the critical role of health literacy in medicine and public health has gained considerable attention [12,13]. The multi-level consequences of low health literacy have been reviewed extensively and include negative health behaviors, reduced utilization of preventive health services, and poorer adherence to therapeutic protocols [14,15]. Data from the most recent National Adult Literacy Survey (2003) indicate that an alarming proportion of US adults are functionally illiterate [16], and there exists evidence connecting low literacy with poorer health-related quality of life [17]. Health literacy is now considered an underlying cause of health disparities and has become a national health priority [18,19].

Although much is known about health literacy in the medical context, little is known about *oral health literacy *(OHL) and its relationship to clinical conditions, patients' subjective assessments, and OHL's perceived impacts on daily life in the community. A working group of the National Institutes of Dental and Craniofacial Research (NIDCR) defined OHL as "the degree to which individuals have the capacity to obtain, process, and understand basic oral health information and services needed to make appropriate health decisions" [20]. Horowitz and Kleinman recently proposed that "oral health literacy is the new imperative for better oral health" as health literacy is now considered a determinant of health [21].

An accumulating body of evidence links low OHL with worse oral health outcomes such as oral health status [22,23], dental neglect [24] as well as sporadic dental attendance [25]. In a investigation among a group of Indigenous Australians, Parker and Jamieson [26] found that although low OHL was not associated with self-reported oral health status, it was associated with increased prevalence of OHIP-14 impacts (proportion of items reported fairly/very often). Noteworthy, in a recent study among child-caregiver dyads in the US, caregivers' OHL modified the association between children's oral health status and child OHRQoL impacts, with low-literacy caregivers reporting less impacts [27].

Previous pilot studies have explored the patterns of association between OHL and measures of OHRQoL using the Test of Functional Health Literacy in Dentistry (TOFHLiD) [28] and the Rapid Estimate of Adult Literacy in Dentistry (REALD-99) [29]. Interestingly, as in the Parker and Jamieson study, Richman and colleagues reported that while OHL was not associated with dental health status, higher OHL scores were significantly associated with less perceived OHIP-14 impacts, indicating better OHRQoL [29].

In the validation study of the short form of the REALD (REALD-30) among patients in a medical clinic setting, Lee *et al *[24] reported an inverse association of REALD-30 with OHIP-14 scores; however, the authors noted that because the data were collected on a convenience sample of health care-seeking subjects, future work is warranted on a larger, more diverse sample, as recommended by the NIDCR proposed research agenda [20]. To this end, the aims of the present study were to investigate the association between OHL and OHRQoL using REALD-30 in a large and more diverse and non-care seeking sample of subjects, and to explore any differences in this association between racial groups.

## Methods

### Study population and recruitment

This investigation relied upon interview data from the Carolina Oral Health Literacy (COHL) Project [30], a study exploring OHL in a low-income population of caregivers in the Women, Infants, and Children's Supplemental Nutrition Program (WIC) in North Carolina (NC). Non-random WIC sites in 7 counties in NC were selected using certain criteria including geographic region, rural/urban makeup, population demographics, active WIC clinics and established working relationships.

Study staff members were deployed in the selected WIC clinics and approached consecutive individuals to ask if they would answer eight questions from the study eligibility screening instrument. Eligibility criteria included being: a) the primary caregiver of a healthy (ASA I or II) and Medicaid-eligible infant/child 60 months old or younger, or expecting a newborn within the next 8 months, b) 18 years or older and c) English-speaking. Caregivers that met these criteria and agreed to participate were accompanied to a private area for a 30-minute in-person interview with one of the two trained study interviewers. Purposeful quota sampling [31] was employed to ensure that minority groups would be well-represented in the study sample. In this approach, individuals in pre-determined minority groups (African Americans and American Indians in the COHL study) are targeted preferentially and recruited into the study until adequate representation in the final sample is achieved. From 1,658 subjects that were screened and determined eligible 1,405 (85%) participated and provided data in the domains of socio-demographic information, dental health and behaviors, OHRQoL, self-efficacy, and OHL. For the current analysis we excluded men (n = 49 or 3.5% of total), Asians (n = 12, or 0.9%), those who did not have English as their primary language at home (n = 79 or 5.6%), and those who had not yet reached age 18 (n = 2 or 0.1%). Therefore, our analytic sample included White, African American (AA) or American Indian (AI) female caregivers, whose primary language was English (N = 1,278).

### Variable Measurements

Additional demographic characteristics included age and education. Age was measured in years and coded as a quintile-categorical indicator variable. Education was coded as a four-level categorical variable where 1: did not finish high school, 2: high school or General Education Diploma (GED), 3: some technical education or some college, 4: college or higher education. Dental attendance was self-reported as the time since the last dental visit and coded as a four-level categorical variable where 1: < 1 year, 2: 12-23 months, 3: 2-5 years, 4: > 5 years or never.

OHRQoL impacts were assessed with the use of the short form of the Oral Health Impact Profile (OHIP-14) index [32]. Consistent with previous investigations [11], three OHIP-14 estimates were derived from subjects' responses: *Severity *(cumulative OHIP-14 score), *prevalence *(proportion of subjects reporting fairly/very often one or more items) and *extent *(number of items reported fairly/very often) of impacts were calculated as measures of OHRQoL. In terms of interpretation, the authors acknowledge Locker's critique that the OHIP may not fully satisfy the criteria for 'quality of life' measures [33], to be consistent with previous publications, however, have adopted the widely used term of OHRQoL in this manuscript.

OHL was measured with the previously validated word recognition test (REALD-30) [23]. The REALD-30 includes 30 words of dental context (e.g. fluoride, plaque, caries, halitosis, temporomandibular, etc.) arranged in order of increasing difficulty. The criteria used to determine word difficulty were based on word length, number of syllables, and difficult sound combinations, as well as results from 10 pre-test interviews that had been conducted prior to the REALD-30 validation study [23]. The study participant is asked to read each word out loud with one point given for each word that is pronounced correctly, resulting in a 0-30 cumulative score where 0: lowest and 30: highest literacy. Although the REALD-30 is a word recognition test and may be capturing only some aspects of literacy skills, it has been shown to be highly correlated with functional health literacy [28] and to possess good psychometric properties [23]. Norms or thresholds for what constitutes "low OHL" have not been established, however in previous investigations [27,34] a threshold of < 13 on the 30-point REALD-30 scale was used to define a "low OHL" group.

### Analytical Strategy

We used bivariate tabular methods to display the distribution of the three OHRQoL estimates (severity, prevalence and extent) by strata of socio-demographic variables. We calculated Spearman's correlation coefficients (*rho*) and 95% confidence limits (CL; obtained with bootstrapping, N = 1,000 repetitions) to quantify the associations between OHL scores and *prevalence*, *severity*, and *extent*.

Although the inverse association between OHL and OHRQoL has been shown in previous investigations [23,26], no information has been reported regarding the shape and gradient characteristics of this relationship. For this reason, we used polynomial smoothing functions (LPSF) and corresponding 95% CL to illustrate the relationship between the OHL scores and OHIP-14 estimates. LPSF are non-parametric and data-adaptive functions [35,36] that are flexible in displaying an association without prior assumptions about its shape, gradient, or monotonicity, while minimizing biases from misspecification that could be introduced by traditional modeling applications. Further, to examine the association between "low" OHL and OHRQoL we used the < 13 REALD-30 score threshold, representing the lowest quartile of the distribution, to define the "low OHL" stratum. We obtained crude and adjusted differences and ratios of OHIP-14 impacts using Poisson models.

**Figure 1:**
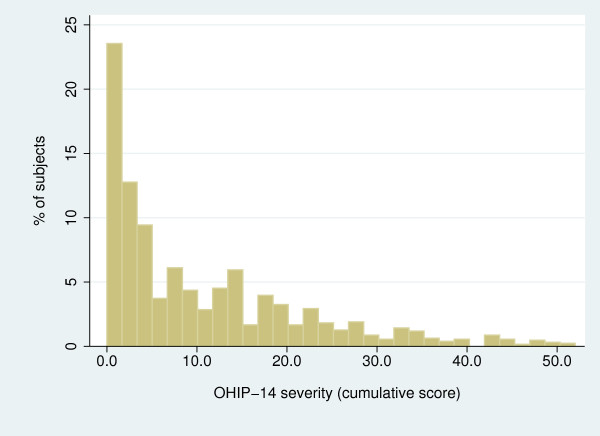
**Distribution of OHIP-14 *severity *(cumulative score) among the female caregivers participating in the COHL study (N = 1,278)**.

Because *severity *is the OHIP-14 estimate that arguably carries the most information (no items or scoring schemes are arbitrarily collapsed) and the entire range of the instrument scale (0-56) [11], we chose this measure for subsequent analytical iterations. To further quantify the association between OHL and *severity*, we used Zero-Inflated Negative Binomial modeling (ZINB). This analytical approach was used because of the distribution characteristics of *severity*, which followed a negative binomial type distribution with "excess zeros" (Figure 1).

The ZINB explicitly specifies two models that are fit simultaneously, one that models the "probability of zero" and one that models the count outcome, using a negative binomial distribution. These models have gained popularity in analyses of count outcomes with high proportion of zeros, but their selection and applicability can be data-specific [37,38]. For this reason and to determine the best fit, we considered other analytical approaches including the negative binomial (NB) and the zero inflated Poisson (ZIP) model. The appropriateness of ZINB *versus *the NB or the ZIP model was tested and confirmed with diagnostic model-fit statistics, using a Vuong test (ZINB favored over NB, P < 0.05) and a likelihood ratio test (ZINB favored over ZIP, P < 0.05) [39].

The exponentiated coefficient of the negative binomial component of the model corresponds to a Prevalence Rate Ratio, which in this analysis we interpret as ratio of reported impacts (problems), or "Problem Rate Ratio" (PRR) as in a previous study [40]. To facilitate interpretation, we report model coefficients that correspond to one standard deviation change in OHL, which in our study was 5.3 units on the 30 unit REALD-30 scale. In other words, the PRR correspond to the change in reported cumulative OHIP-14 impacts that is associated with one standard deviation change in REALD-30 (expressed as ratio). Inclusion of confounders in the Poisson and the ZINB models was determined by likelihood ratio tests, comparing nested (reduced) models with the referent (full) model using a criterion of P < 0.1. Interpretation of the model coefficients was based on effect estimation rather than hypothesis testing [41].

We employed three (race-specific) multivariate models to explore the possible heterogeneity of the association between OHL and OHRQoL between racial groups. Consistent with our aims, we considered race as an *a priori *modifier of the examined association and therefore, these three models were identical to the "main effects" model but were restricted to strata of Whites, AAs and AIs. To determine whether race modified the association between literacy and quality of life, we compared these model-obtained race-specific estimates of the association between OHL and *severity*. The rationale for conducting comparisons of stratum-specific estimates as opposed to testing the hypothesis in the context of statistical interaction is based on the fact that the former approach does not assume covariate effect-homogeneity across racial groups. This could be a source of non-negligible bias when quantifying a weak main effect (e.g. OHL) in the presence of strong confounders (e.g. education), unless all potential interaction terms are included. To that end, we first conducted a global Wald X^2 ^test of homogeneity or "a common PRR across racial groups" using a conservative criterion of P < 0.2. We further examined *post hoc *differences in estimates between racial groups by calculating three pairwise homogeneity Z-scores (Z_homog_) using the formula: Z_homog_= |b_x_-b_y_|/(se_x_^2^+se_y_^2^)^1/2^, where b_x/y/z _and se_x/y/z _are the ZINB model-obtained race-specific coefficients and standard errors respectively [42]. Two-tailed P-values corresponding to the Z-scores were obtained using the normal distribution function of the Stata 12.0 (StataCorp LP, College Station, TX) statistical program. A P < 0.05 criterion was used for the pairwise tests.

## Results

The demographic characteristics of our final analytic sample (N = 1,280) with corresponding OHIP-14 *prevalence*, *extent*, and *severity *scores are presented in Table 1. Participants' mean age in years was 26.6 (median = 25). Sixty percent had a high school education or less. Seventy-five percent reported a dental visit within the last two years.

**Table 1:** Distribution of oral health-related quality of life (OHRQoL) measures [OHIP-14 estimates and corresponding 95% confidence limits (CL)] by demographic characteristics among the Carolina Oral Health Literacy study participants (N = 1,278)

			**Subjective oral health impacts estimates (OHIP14)**		
	**N**	**(%)**	**Prevalence** **(95% CL)**	**Severity** **(95% CL)**	**Extent** **(95% CL)**
	
**Race**					
White	503	39	36.6 (32.4, 40.8)	10.6 (9.6, 11.6)	1.39 (1.15, 1.62)
African American	522	41	34.7 (30.6, 38.8)	10.4 (9.4, 11.3)	1.24 (1.04, 1.45)
American Indian	253	20	39.1 (33.1, 45.2)	11.2 (9.8, 12.6)	1.53 (1.19, 1.87)
**Education**					
Less than high school	305	24	49.5 (43.9, 55.2)	13.6 (12.1, 15.0)	2.10 (1.74, 2.45)
High school diploma/GED	479	37	35.1 (30.8, 39.4)	10.3 (9.3, 11.3)	1.23 (1.01, 1.45)
Some technical or college	429	34	31.5 (27.1, 35.9)	9.4 (8.5, 10.4)	1.10 (0.88, 1.31)
College or higher	65	5	15.4 (6.4, 24.4)	7.1 (4.9, 9.2)	0.45 (0.15, 0.74)
**Dental attendance**					
< 12 months	726	57	34.7 (31.2, 38.2)	10.4 (9.6, 11.2)	1.30 (1.12, 1.48)
12-23 months	217	17	31.3 (25.1, 37.6)	9.5 (8.0, 11.0)	1.24 (0.91, 1.57)
2-5 years	177	14	45.8 (38.4, 53.2)	11.2 (9.5, 12.9)	1.52 (1.16, 1.88)
> 5 years	151	12	39.9 (32.2, 47.6)	12.6 (10.7, 14.4)	1.58 (1.11, 2.04)
**Age **(years; quintiles)		Mean(SD)			
Entire sample	1,278	26.6(6.9)			
*Q1 *range: 18.0, 20.9	256	19.6(0.8)	28.9 (23.3-34.5)	8.3 (7.1-9.6)	1.04 (0.77, 1.32)
*Q2 *range: 20.9, 23.4	256	22.1(0.7)	40.6 (34.6-46.7)	11.2 (9.8-12.5)	1.47 (1.16, 1.79)
*Q3 *range: 23.4, 26.5	255	24.8(0.9)	34.5 (28.6-40.4)	10.4 (9.1-11.7)	1.22 (0.92, 1.53)
*Q4 *range: 26.5, 30.9	256	28.6(1.3)	37.1 (31.2-43.1)	10.8 (9.5-12.1)	1.35 (1.04, 1.66)
*Q5 *range: 30.9, 65.6	255	37.7(6.1)	40.4 (34.3-46.5)	12.5 (10.8-14.1)	1.69 (1.32, 2.06)

The OHL score was distributed normally [30] with a mean (SD) REALD-30 of 15.8 (5.3), with 25% of participants (N = 316) scoring less than 13, classified as "low OHL". Pronounced OHL gradients were noted relative to education as follows: less than high school--13.0 (4.8), high school or GED--15.0 (4.9), some technical or college--18.0 (4.7) and college degree or higher--20.1 (4.8). Differences by race were also evident: whites--17.4 (4.9), AA--15.3 (5.1), AI--13.7 (5.3). The mean OHIP-14 *severity *and *extent *scores were 10.6 (95% CI = 10.0, 11.2) and 1.35 (95% CI = 1.21, 1.50), respectively. Thirty-seven percent reported at least one oral health impact fairly or very often (*prevalence*), while AIs had the highest *severity *score. A strong gradient was found with decreasing age and OHIP-14 scores. Some age and racial differences were noted, with older subjects and AIs reporting more impacts.

**Figure 2:**
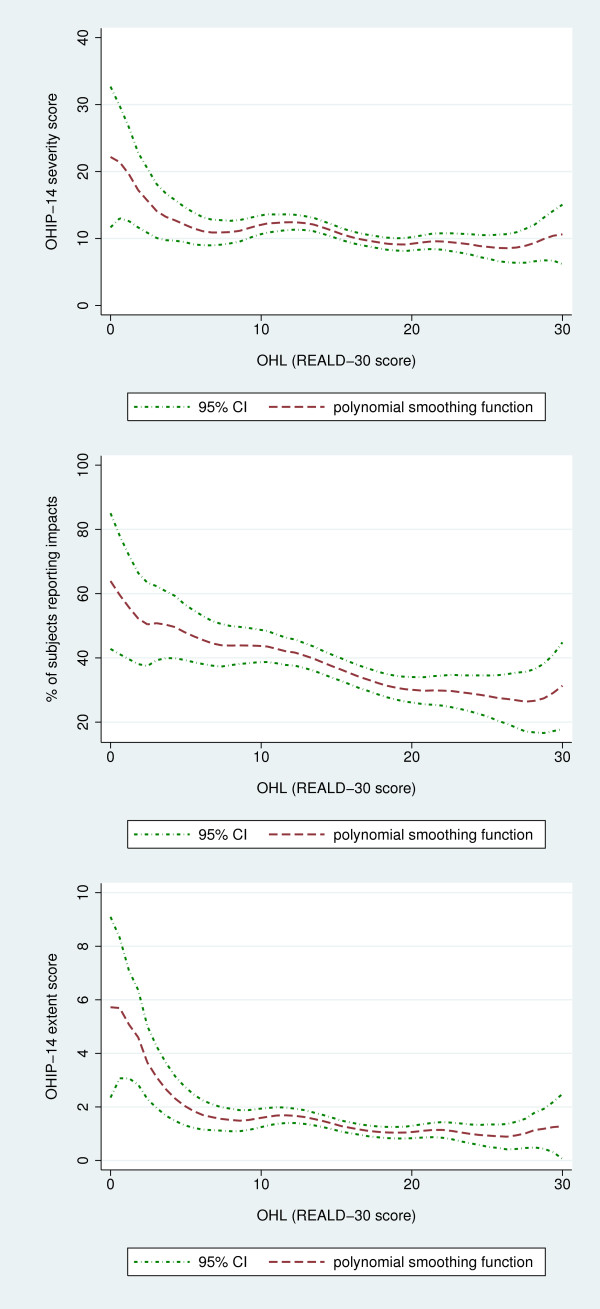
**Relationship between OHL and oral health related quality of life estimates [OHIP-14 severity (a), prevalence (b) and extent (c)] illustrated by polynomial smoothing functions and corresponding 95% confidence limits, among the female caregivers participating in the COHL study (N = 1,278)**.

OHL showed weak correlations with all three OHIP-14 estimates: *prevalence rho*= -0.14 (95% CI = -0.20, -0.08), *extent rho *= -0.14 (95% CI = -0.19, -0.09), and *severity rho *= -0.10 (95% CI = -0.16, -0.05). These bivariate associations are illustrated in Figures 2a, b, and 2c with local polynomial smoothing functions and 95% confidence intervals. In these illustrations the inverse, non-linear association between OHL and the OHRQoL estimates was evident. Although the negative gradient was more apparent for *prevalence*, the inverse relationship of all three OHRQoL measures with OHL was more "profound" at the lower end of the OHL range. This was confirmed by the contrast of the "low" versus the "high OHL" group (Table 2), where the former group had consistently worse OHRQoL estimates. "Low OHL" was associated with significant absolute and relative increases in all OHRQoL dimensions, with relative prevalence estimates ranging from +11% for severity to +34% for extent.

**Table 2:** Oral health-related quality of life (OHRQoL) differences [mean difference and prevalence ratios (PR) with corresponding 95% confidence limits (CL)] between participants with "low" (< 13 REALD-30; referent category) and "high" (≥ 13 REALD-30) oral health literacy in the Carolina Oral Health Literacy study (N = 1,278)

	**"Low" literacy** **(< 13 REALD-30)**	**"High" literacy** **(≥ 13 REALD-30)**	**Difference^1 ^[mean (95% CL)]**		**Prevalence Ratio^1 ^[(PR (95% CL)]**
	**N = 316 (25%)**	**N = 962 (75%)**	**Crude**	**Adjusted**^**2**^	Adjusted^2^
			
**OHRQoL** (OHIP-14 estimates)					
*Prevalence*	45.3 (39.7, 50.8)	33.4 (30.4, 36.4)	11.9 (0.04, 0.20)	7.4 (-1.4, 16.2)	1.17 (1.00, 1.37)
*Severity*	12.4 (11.0, 13.8)	10.1 (9.4, 10.7)	2.3 (1.9, 2.8)	1.2 (0.7, 1.6)	1.11 (1.07, 1.16)
*Extent*	1.87 (1.52, 2.22)	1.19 (1.04, 1.33)	0.68 (0.52, 0.85)	0.36 (0.19, 0.54)	1.34 (1.20, 1.50)

1: Mean differences and ratios of OHIP-14 impacts were calculated using the "high literacy" category as referent.

2: Adjusted differences and ratios were obtained using a Poisson model controlling for race, age, education level and dental attendance.

Multivariate analysis adjusting for age, race, and education revealed that the weak inverse association between OHL and *severity *across the entire sample persisted: PRR = 0.91 (95% CL = 0.86, 0.98). Table 2 presents estimates obtained from the stratified (race-specific) multivariate models, where: Whites--PRR = 1.01 (95% CL = 0.91, 1.11), AA--PRR = 0.86 (95% CL = 0.77, 0.96) and AI--PRR = 0.92 (95% CL = 0.80, 1.05). By comparing these estimates *ensemble *we rejected the assumption of homogeneity (Wald X^2 ^= 4.6; degrees of freedom = 2; P < 0.2). Subsequent pairwise comparisons of the race-specific estimates confirmed that the measures of association among AAs and Whites departed from homogeneity (Z_homog _= 2.06; P < 0.05). In fact, no association between OHL and OHIP-14 *severity *was found among Whites whereas weak associations were found among AAs and AIs.

## Discussion

This investigation provides the first report of the association between OHL and OHRQoL (as measured by OHIP-14) in a multi-racial community-based sample. This study was restricted to a non-probability sample of low-income female caregivers participating in the WIC program in NC; however, we believe that this homogeneity is advantageous because strong income-gradients have been identified in oral health impacts on the population level [43,44]. Moreover, recruitment of subjects from a non-dental clinical environment reduces the potential for selection bias and, within the limitations of the sampling procedures and target population, increases the generalizability of our findings. It is noteworthy but not surprising that the OHL levels in this study were considerably lower than those reported for dental patients seeking care in private practice [REALD-30 (SD): 23.9 (1.3)] [22] or a dental school setting [20.7 (5.5)] [45], and comparable to those found among a community-based sample of indigenous Australians [15.0 (7.8)] [26].

It has been acknowledged that minority individuals and those towards the lowest end of the literacy distribution may be underrepresented in oral health research [46] and this can be even more exacerbated in literacy investigations. Interestingly, the most profound negative gradients between OHL and OHRQoL measures were observed at the lower end of the OHL spectrum, with subjects scoring < 13 on the 30-point REALD-30 scale reporting significantly more OHRQoL impacts *versus *those with higher literacy. This finding is consistent with conceptual frameworks that consider skills such as conceptual knowledge and OHL as pre-requisites of appropriate decision-making [47]. It is likely that OHL exerts strong influences on oral health-related outcomes when below a certain threshold, but it may be a less impactful determinant at higher levels.

The high representation of AAs and AIs that were enrolled in COHL offered us an opportunity to examine for any underlying heterogeneity in the association of OHL with SOH between racial groups. We found a weak negative association between OHL and OHIP-14 *severity *for AAs and AIs, but not Whites. While AAs have been shown to report worse OHIP scores in the US [10] and patterns of OHRQoL changes have been shown to differ by race [48,49], this finding warrants further investigation; race may be a proxy of unmeasured mediating factors between OHL, oral health status, and perceived impacts [50]. The fact that the dimensionality of OHRQoL [8] may differ between diverse populations or ethnic groups may amplify this phenomenon; therefore, we acknowledge the limitation of our analytical sample that was restricted to low-income WIC-participating female caregivers. Replication of our main as well as race-specific findings should be undertaken on a population-based representative sample.

Lawrence *et al *[51] recently demonstrated that OHIP-14 scores show good correlation with clinical oral health status, independent of gender and socioeconomic inequalities in oral health. Among our community-based caregivers, the *prevalence *of oral health impacts (36.5%) was higher compared to nationally representative samples from other studies including the US (15.3%) [10], Australia (dentate subjects-18.2%), United Kingdom (dentate subjects-15.9%) [11] and New Zealand (23.4%) [51]. However, the *extent *and *severity *estimates reported here are lower compared to these samples. One possible interpretation of this finding is that our study group was limited to young, low-income, poorly educated, WIC participants with relatively low education. The young mean age (26.6 years) may explain the low *severity *and *extent *estimates while the low-income and low-education level status may explain the high *prevalence *of at least one impact reported as fairly/very often.

Considering the high *prevalence *of impacts revealed in the study population, the significance of lower OHL is demonstrative. Using our "main effects" model coefficients, we estimate that a one standard deviation increase in OHL (5.3 REALD-30 units) corresponds to a 9% decrease in OHIP-14 *severity *[PRR (95% CL) = 0.91 (0.86, 0.98)], whereas (using race-specific estimates from Table 3) this decrease is more pronounced (14%) among AA [PRR (95% CL) = 0.86 (0.77, 0.96)]. On the other hand, this finding provides a foundation to consider interventions to enhance OHL, or rather improve the readability of written materials and accessibility to dental services to an appropriate literacy level [30]. It remains uncertain whether improvement in OHL is feasible and if so, whether this would lead to better oral health status and subjective oral health. Although education and income arguably remain the strongest correlates of oral health and disease, and literacy is one of numerous other distal determinants, OHL may be part of causal mechanisms that lead to worse oral health [21]. Accumulating evidence linking poor OHL with adverse oral health outcomes among caregivers [24] and their young children [27,34] supports the introduction and implementation of rapid OHL screening tools [52] in clinical practice, dental research and public health surveillance. Moreover, we suggest that more studies exploring the association between OHL and OHRQoL be undertaken in multi-racial community based samples to confirm or reject this study's finding of effect measure modification by race.

**Table 3:** Adjusted^1 ^'problem' rate ratios (PRR) of OHIP-14 severity (cumulative score) corresponding to one standard deviation change in OHL (5

	**PRR^2^**	**95% CL**
	
Entire sample	0.91	0.86, 0.98
**Race**		
White	1.01	0.91, 1.11
African American	0.86	0.77, 0.96
American Indian	0.92	0.80, 1.05

1: Zero-inflated negative binomial model, including terms for age, education level and dental attendance.

2: Estimates corresponds to the relative change in OHIP-14 cumulative score for one standard deviation increase in OHL.

## Conclusions

We found a high prevalence of perceived oral health impacts in this sample of low-income female WIC participants. Although the inverse association between OHL and OHRQoL across the entire sample was weak, subjects in the "low" OHL group reported significantly more OHRQoL impacts *versus *those with higher literacy. Within the limitations of our study among low-income female caregivers, our findings indicate that the association between OHL and OHRQoL appears to be modified by race.

## Competing interests

The authors declare that they have no competing interests.

## Authors' contributions

KD conducted the data analysis and prepared the first draft of the manuscript. JL conceived the study, overviewed the data analysis, contributed to the interpretation of results and assisted in preparation of the first draft of the manuscript. ADB participated in data collection, and critically revised the manuscript. WFV contributed to the interpretation of results and critically revised the manuscript. All authors read and approved the final manuscript.
